# Development and validation of a novel nomogram model for predicting delayed graft function in deceased donor kidney transplantation based on pre-transplant biopsies

**DOI:** 10.1186/s12882-024-03557-3

**Published:** 2024-04-19

**Authors:** Meihe Li, Xiaojun Hu, Yang Li, Guozhen Chen, Chen-guang Ding, Xiaohui Tian, Puxun Tian, Heli Xiang, Xiaoming Pan, Xiaoming Ding, Wujun Xue, Jin Zheng

**Affiliations:** Department of Renal Transplantation, Nephropathy Hospital, The First Affiliated Hospital of Xi’an Jiaotong University, 710061 Xi’an, Shaanxi China

**Keywords:** Kidney transplantation, Delayed graft function, LASSO regression, Nomogram, Pre-transplant biopsy

## Abstract

**Background:**

Delayed graft function (DGF) is an important complication after kidney transplantation surgery. The present study aimed to develop and validate a nomogram for preoperative prediction of DGF on the basis of clinical and histological risk factors.

**Methods:**

The prediction model was constructed in a development cohort comprising 492 kidney transplant recipients from May 2018 to December 2019. Data regarding donor and recipient characteristics, pre-transplantation biopsy results, and machine perfusion parameters were collected, and univariate analysis was performed. The least absolute shrinkage and selection operator regression model was used for variable selection. The prediction model was developed by multivariate logistic regression analysis and presented as a nomogram. An external validation cohort comprising 105 transplantation cases from January 2020 to April 2020 was included in the analysis.

**Results:**

266 donors were included in the development cohort, 458 kidneys (93.1%) were preserved by hypothermic machine perfusion (HMP), 96 (19.51%) of 492 recipients developed DGF. Twenty-eight variables measured before transplantation surgery were included in the LASSO regression model. The nomogram consisted of 12 variables from donor characteristics, pre-transplantation biopsy results and machine perfusion parameters. Internal and external validation showed good discrimination and calibration of the nomogram, with Area Under Curve (AUC) 0.83 (95%CI, 0.78–0.88) and 0.87 (95%CI, 0.80–0.94). Decision curve analysis demonstrated that the nomogram was clinically useful.

**Conclusion:**

A DGF predicting nomogram was developed that incorporated donor characteristics, pre-transplantation biopsy results, and machine perfusion parameters. This nomogram can be conveniently used for preoperative individualized prediction of DGF in kidney transplant recipients.

**Supplementary Information:**

The online version contains supplementary material available at 10.1186/s12882-024-03557-3.

## Introduction

Delayed graft function (DGF) is a common complication in organ transplantation. DGF is a form of acute renal failure [1] that can result in increased allograft immunogenicity, leading to subsequent acute rejection and graft failure. As reported earlier [2], DGF is remarkably associated with long-term dysfunction. There is no consensus in the literature about how to define DGF. The straightforward United Network for Organ Sharing definition of DGF is the need for at least one dialysis treatment in the first week after transplantation (classical DGF) [3]. According to the 2018 Annual Report of the Organ Procurement and Transplantation Network/Scientific Registry of Transplant Recipients (OPTN/SRTR) [4] and the 2016 Annual Report of the European Renal Association-European Dialysis and Transplant Association (ERA-EDTA) Registry [5], the incidence of DGF ranged between 20% and 30% in the United States and almost 50% in Europe.

DGF can lead to several health-related consequences. It not only increases the risk of graft failure but also prolongs hospitalization, thereby increasing healthcare-related expenditure. Additionally, a high rate of mortality is reported in recipients diagnosed to have DGF [6].

DGF is a multifactorial event. The risk factors of DGF include donor and recipient characteristics, pre-transplantation biopsy results [7], and machine perfusion parameters [8]. Irish et al. developed a primary DGF prediction model based on a nomogram by considering donor and recipient clinical factors alone [9]. The incorporation of machine perfusion parameters and pre-transplantation biopsy results into a clinical variable-based predictive model can improve its prognostic performance. Traditional regression models such as logistic regression, however, are limited due to overfitting when several covariates are included [10]; this implies that regression models fit the training cohort well, but they cannot be generalized to sufficiently reflect real-world cases. Moreover, variable selection is important if a high-dimensional feature exists [11].

In the least absolute shrinkage and selection operator (LASSO) regression model, the estimates of the regression coefficients are sparse, which implies that many components have exactly zero values. Thus, LASSO automatically deletes unnecessary covariates. LASSO has many desirable properties for regression models with several covariates.

The present study aimed to develop and validate a comprehensive predictive model to better stratify kidney transplant recipients according to DGF risk. We used the LASSO-logistic regression method to select suitable covariates from a vast amount of clinical and histological data obtained from pre-transplantation biopsy of kidney allografts. We also investigated the gain in the accuracy of the comprehensive nomogram model by incorporating histological signature and clinical risk factors for the preoperative prediction of DGF.

## Materials and methods

### Patients and ethical approval

This retrospective study was approved by the Ethics Committee of the First Affiliated Hospital of Xi’an Jiaotong University (Shaanxi, China), No. XJTU1AF2015LSL − 058. All participants signed the informed consent form. The study protocol complied with the *Declaration of Helsinki* and *Istanbul* principles. Kidneys for transplantation were obtained from the Coordination Group of Shaanxi Red Cross Organization and harvested by the Organ Procurement Organization (OPO). No organs were harvested from executed prisoners. The immediate relatives of the donors voluntarily offered organ donation. Organ allocation was performed based on the China Organ Transplant Response System, and the process was kept as double-blind between donors and recipients.

### Immunosuppressive regimen

On the day of transplantation surgery, all recipients were intravenously administered induction therapy by using rabbit anti-thymocyte globulin (r-ATG, 50 mg or 75 mg), ATG-Fresenius (ATG-F, 200–300 mg), or basiliximab (40 mg). The dose of r-ATG or ATG-F was tapered until discontinuation on postoperative day 5, and basiliximab 40 mg was provided again on postoperative day 4. Since the first day after transplantation, each recipient received the triple immunosuppressive regimen consisting of a mycophenolic acid drug (MPA: enteric-coated mycophenolate sodium or mycophenolate mofetil), a calcineurin inhibitor (CNI: tacrolimus or cyclosporine A), and prednisone.

### Study design

The inclusion criteria for donors were as follows: (i) had clear identity and met the medical and ethical conditions for organ transplantation; (ii) had no history of kidney disease, drug abuse, and active infection diseases such as HIV and HBV; (iii) had no history of diabetes mellitus with severe complications; and (iv) had no history of malignant tumor.

Recipients were excluded if: (1) recipients who developed graft failure within 48 h of the transplant operation; (2) had a positive cross match or positive panel-reactive antibody (over 30%); (3) had an active infection, hepatitis, or abnormal hepatic function; or (4) had leukopenia (leukocytes < 3000/mm^3^), thrombocytopenia (platelets < 100,000/mm^3^), or severe anemia (hemoglobin < 60 g/L); (4) recipients who received re-transplantation or dual kidneys; (5) children’s kidney (6) recipients who received combined liver transplant; (7) Recipients with body mass index (BMI) < 28 kg/m^2^.

The donor scoring system included the donor’s age, primary disease, sCr levels prior to organ recovery, history of hypertension, CPR incidence and hypotension duration. The value of donor clinical scores in predicting graft performance was previously developed and validated from a thousand-patient cohort at our center [12]. Donors above 16 years of age with confirmed identity; with no history of kidney diseases, diabetes, drug abuse, and uncontrollable psychotic symptoms; who were not actively infected with hepatitis B and C viruses, human immunodeficiency virus, bacteria, and fungi; and in whom the isolated renal had a warm ischemia time (WIT) < 30 min and a cold ischemia time (CIT) < 12 h were included in the study. At least one kidney from each donor was used for single renal transplantation. Executed prisoners were excluded from the study.

DGF was considered as the primary outcome of this study, and it was defined as the need for dialysis [13] in the first week after kidney transplant surgery. Two independent datasets were used in this study, including the training cohort and the validation cohort. The training cohort was used to construct the predictive model and included 492 kidney transplant recipients between May 2018 and December 2019. The independent validation cohort was used to test the predictive model and included 105 recipients who underwent transplantation surgery between January 2020 and April 2020.

### Variables and samples

Three transplant surgeons independently assessed the clinical characteristics of the included donors and recipients. The donor clinical characteristics included age, gender, body mass index (BMI), ABO blood type, cause of death (cardiac death), hypertension history (presence or absence), hypotension procedure (systolic pressure < 100 mmHg, presence or absence), cardiopulmonary resuscitation (CPR) procedure (presence or absence), terminal renal function (including serum creatinine (SCr) and blood urea nitrogen (BUN) levels), and urine volume. The recipient clinical characteristics included age, gender, BMI, primary disease, ABO blood type, dialysis method (hemodialysis or peritoneal dialysis) and duration, human leukocyte antigen (HLA) mismatch, pre-transplant panel reactive antibody (PRA) level (positive or negative), and type of induction therapy (r-ATG, ATG-F, or basiliximab).

After organ procurement, the kidneys were preserved by static cold storage (SCS) or hypothermic machine perfusion (HMP). HMP was performed using the LifePort Kidney Transporter machine (Organ Recovery Systems, Chicago, IL, USA). The initial pump pressure was set as 30–40 mmHg. The machine recorded the following five parameters: pressure, temperature, resistance, flow rate, and duration. The following characteristics of organ transport were recorded by OPO and transplant surgeons: transport method (SCS or HMP), machine perfusion parameters (initial and terminal pressure, flow rate, and resistance), cold ischemic time (CIT), and warm ischemic time (WIT).

Pre-implantation biopsies were performed by the transplant surgeon by using a 16G Bard needle. One sample was obtained from each kidney; fixed in formaldehyde; embedded in paraffin; sectioned; and stained with hematoxylin and eosin, periodic acid-Schiff, Masson’s trichrome, and silver methenamine. Each section was evaluated by two pathologists independently. Light microscopy was performed, and Banff 2022 classification [14] was used to evaluate chronic histopathological changes in the kidney. Acute changes, including acute tubular injury (ATI) and arteriolar smooth muscle vacuolar degeneration, were also noted. Each chronic or acute lesion was recorded on the scale of 0–3 points according to the degree of severity. Remuzzi score [15] and Banff score were calculated according to semi-quantitative chronic histopathological changes. All biopsies were performed pre-implantation and after conducting HMP/SCS.

### Development of a nomogram model

Continuous variables are reported as mean ± SD (standard deviation), and categorical variables are reported as primary frequencies (percentages). HMP parameters, histological lesions, and donor and recipient clinical characteristics were analyzed using the Mann-Whitney *U* test or the chi-square test to determine significant differences between the DGF and non-DGF groups. Significant variables were included in the multivariate model. However, because of the small number of events relative to the number of factors and to obtain an optimal model with as few factors as possible, we used L1-penalized LASSO regression for multivariate analysis [16]; this was the first-step variable selection process. This logistic regression model penalizes the absolute size of the regression coefficients according to the lambda value. With larger penalties, the estimates of weaker factors shrink toward zero; consequently, only the strongest predictors remain in the model, and weaker predictors are excluded. To avoid overfitting models to idiosyncratic relationships in the training cohorts, the variable selection process used 10-fold cross-validation to select the optimal level of tuning or penalization, as measured by the Bayesian information criterion. A nomogram model was developed using variables with nonzero coefficients through multivariate logistic regression.

### Validation and performance of the nomogram

Calibration curves were plotted to calibrate the prediction nomogram, accompanied with the Hosmer-Lemeshow (H-L) test. A nonsignificant test result (*P* > 0.05) implies that the calibration of the model is inaccurate. To assess the discrimination of the prediction nomogram, a receiver operating characteristic curve (ROC) was plotted, and area under the curve (AUC) values were determined. Calibration curves and ROC of the validation cohort were obtained to validate this nomogram.

Decision curve analysis (DCA) was performed to determine the clinical usefulness of the nomogram by quantifying the net benefits at different threshold probabilities in the validation cohort [17].

### Pre-transplant biopsies

Pre-implantation biopsies were performed by the transplant surgeon using a 16-g Bard needle. Two biopsies were performed for each donor kidney. One piece of tissue was embedded for immunofluorescence staining, including IgA, IgM, IgG, C3, C1q, and fibrin-related antigens. Another biopsy tissue was fixed with formaldehyde, embedded in paraffin, sectioned and stained with hematoxylin and eosin, periodic acid Schiff, Masson trichrome, and hexamine silver. The donor kidney biopsy tissue contained at least 25 glomeruli, and Remuzzi score was immediately performed according to the rapid biopsy results [15]. Remuzzi’s method was used to evaluate the chronic histopathological changes of the donor kidney, and the ATI of the donor kidney was evaluated. According to Remuzzi scoring criteria, the degree of glomerular sclerosis, renal tubular atrophy, interstitial fibrosis and arterial lumen stenosis of the donors were evaluated by pathologists with a score of 0–3. All biopsies were performed before transplantation, but histopathological diagnoses were determined after transplantation to avoid potential selection bias based on histopathological findings.

### Statistical analysis

Statistical analysis was conducted using R version 4.0.0 (www.Rproject.org) and GraphPad Prism v9.0. LASSO logistic regression was performed using the “glmnet” package. The packages “rms,” “pROC,” and “DecisionCurve” were used to plot the nomogram, ROC and AUC, and DCA, respectively. The H-L test was performed using the “generalhoslem” package. The reported significance levels were two-sided and set at 0.05.

## Results

### Donor and recipient clinical characteristics

The development cohort comprised 492 kidney transplant recipients; of these 492 recipients, 96 (19.51%) developed DGF after transplant surgery. Table 1 shows the baseline characteristics of recipients and univariate analysis results. All recipients included those who accepted kidney transplant surgery for the first time. The PRA level alone was significantly different between DGF and non-DGF recipients (*P* = 0.028). No significant differences were observed between the DGF and non-DGF groups in terms of recipient age, gender, BMI, primary disease, blood type, pre-transplant dialysis duration, HLA mismatch, SCr level, and perioperative induction therapy. Table S2 shows the clinical characteristics of the validation cohort.

**Table 1:** Comparison of recipient characteristics between the DGF and non-DGF groups in the development cohort

Recipient characteristics	Non-DGF Group	DGF Group		*P* value
	*n* = 396	*n* = 96		
Age (years)	36.08 ± 9.61	37.66 ± 8.57		0.142
Male, n (%)	275 (69.4%)		72 (75.0%)	0.319
BMI (kg/m^2^)	20.10 ± 1.18		19.98 ± 0.79	0.798
**Primary disease, n (%)**				0.895
Chronic glomerulonephritis	283 (71.5%)		75 (78.1%)	
IgA nephropathy	56 (14.1%)		10 (10.4%)	
Diabetic nephropathy	10 (2.5%)		2 (2.1%)	
Polycystic kidney	18 (4.5%)		4 (4.2%)	
Purpura nephritis	9 (2.3%)		4 (4.2%)	
Others	20 (5.1%)		1 (1.0%)	
Hemodialysis (before transplant), n (%)	333 (84.1%)		74 (77.9%)	0.401
Dialysis duration (months)	19.71 ± 19.49		24.04 ± 26.80	0.075
HLA mismatches	2.0 (1.0, 2.0)		2.0 (1.0, 2.0)	0.337
PRA positive, n (%)	59 (14.8%)		6 (6.3%)	**0.028**
Pre-transplant serum creatinine (µmol/L)	892.41 ± 254.83		938.02 ± 244.74	0.114
**Induction therapy, n (%)**				0.581
r-ATG	255 (64.6%)		62 (64.6%)	
ATG-F	57 (14.4%)		18 (18.8%)	
Basiliximab	58 (14.7%)		10 (10.4%)	
Others	26 (6.56%)		6 (6.3%)	

Data are expressed as mean ± SD, n (%), or median (interquartile range). DGF, delayed graft function; BMI, body mass index; HLA, human leukocyte antigen; PRA, panel reactive antibody; r-ATG, rabbit anti-thymocyte globulin; ATG-F, anti-thymocyte globulin Fresenius

The baseline information on the donors is summarized in Table 2. A total of 266 donors were included in the development cohort. Overall, the mean age (SD) of donors in the cohort was 50.82 ± 12.24 years; 207 donors (77.8%) were males. Trauma (37.2%), and cerebrovascular accident (54.1%) were the main reasons of death of the donors. The mean (SD) SCr level of the donors was 112.45 ± 77.70 µmol/L. Protein in the urine of 16 donors (6.0%) was detected by urine tests before procurement surgery. Univariate logistic regression analysis showed that donor hypertension (odds ratio [OR] = 1.22, 95% confidence interval [CI]: 1.01–1.37, *P* < 0.001), CPR (OR = 2.68, 95% CI: 1.00–7.18, *P* = 0.049), and terminal SCr level (OR = 1.01, 95% CI: 1.00–1.01, *P* = 0.002) significantly influenced DGF onset.

**Table 2:** Donor characteristics

Donor characteristics	Parameter	*P* value	Univariate logistic regression OR (95%CI)
	*n* = 266		
Age (mean ± SD, years)	50.82 ± 12.24	0.654	
Male/female ratio	207/59 (77.8%)	0.768	
BMI (mean ± SD, kg/m^2^)	22.91 ± 2.90	0.542	
**Cause of death (n, %)**			
Trauma	99 (37.2%)		
Cerebrovascular disorders	144 (54.1%)		
Hypoxic ischemic encephalopathy Tumor	13 (4.9%)		
Others	10 (3.7%)		
History of hypertension (n, %)	167 (62.7%)	< 0.001	1.22 (1.01–1.37)
Terminal sCr (mean ± SD, µmol/L)	112.45 ± 77.70	0.002	1.01 (1.00–1.01)
CPR	50 (18.9%)	0.049	2.68 (1.00–7.18)

Data are expressed as mean ± SD, n (%), or median (interquartile range). BMI, body mass index; CPR, cardiopulmonary resuscitation; sCr: serum creatinine

### HMP parameters

A total of 458 kidneys (93.1%) were preserved by HMP. Compared to SCS, HMP did not significantly reduce DGF incidence (SCS 19.8% vs. HMP 14.7%, *P* = 0.611). HMP parameters consisted of initial and terminal perfusion pressure (mmHg), perfusion flow (mL/min), and perfusion resistance (mmHg.min.mL^− 1^). As shown in Table 3, all parameters exhibited significant differences between the DGF and non-DGF groups.

**Table 3:** Comparison of HMP parameters between the DGF and non-DGF groups

Parameters	Non DGF group	DGF group	P value
**Initial stage**			
Pressure (mmHg)	34.85 ± 3.74	36.49 ± 3.74	**< 0.001**
Flow (mL/min)	90.43 ± 18.08	84.31 ± 19.09	**0.004**
Resistance (mmHg.min.mL^− 1^)	0.37 ± 0.11	0.42 ± 0.14	**< 0.001**
**Terminal stage**			
Pressure (mmHg)	29.87 ± 6.40	32.46 ± 5.63	**< 0.001**
Flow (mL/min)	108.26 ± 16.54	100.99 ± 19.09	**< 0.001**
Resistance (mmHg.min.mL^− 1^)	0.24 ± 0.09	0.29 ± 0.11	**< 0.001**

### Pre-transplant biopsies

Chronic and acute lesions in pre-transplant biopsies were assessed through semi-quantitative approaches. Table 4 shows the distribution of pathological lesions between the DGF and non-DGF groups. Except for arterial hyaline degeneration, the distribution of all chronic and acute pathological lesions showed significant differences between the DGF and non-DGF groups. The distribution of arterial hyaline degeneration was not significantly different between the DGF and non-DGF group (*P* = 0.541).

**Table 4:** Pre-transplant biopsy results in the DGF and non-DGF groups

Lesions in pre-transplant biopsy	Degree	Non DGF group	DGF group	P value
**Chronic lesions**				
Glomerular sclerosis	None	257 (65.1%)	40 (41.7%)	**< 0.001**
	Mild	134 (33.9%)	51 (53.1%)	
	Moderate	4 (1.0%)	5 (5.2%)	
	Severe	0 (0.0%)	0 (0.0%)	
Tubular atrophy	None	256 (64.8%)	40 (41.7%)	**< 0.001**
	Mild	135 (34.2%)	27 (28.1%)	
	Moderate	4 (1.0%)	0 (0.0%)	
	Severe	0 (0.0%)	0 (0.0%)	
Interstitial fibrosis	None	257 (65.1%)	40 (41.7%)	**< 0.001**
	Mild	134 (33.9%)	51 (53.1%)	
	Moderate	4 (1.0%)	5 (5.2%)	
	Severe	0 (0.0%)	0 (0.0%)	
Arterial intimal hyperplasia	None	204 (51.8%)	29 (30.2%)	**< 0.001**
	Mild	146 (37.1%)	40 (41.7%)	
	Moderate	43 (10.9%)	27 (28.1%)	
	Severe	1 (0.3%)	0 (0.0%)	
Mesangial matrix hyperplasia	None	284 (75.1%)	41 (46.6%)	**< 0.001**
	Mild	83 (22.0%)	40 (45.5%)	
	Moderate	11 (2.9%)	5 (5.7%)	
	Severe	0 (0.0%)	2 (2.3%)	
Arterial hyaline degeneration	None	238 (60.4%)	52 (54.2%)	0.541
	Mild	72 (18.3%)	23 (24.0%)	
	Moderate	82 (20.8%)	20 (20.8%)	
	Severe	2 (0.5%)	1 (1.0%)	
**Acute lesions**				
Acute tubular injury	None	0 (0.0%)	0 (0.0%)	**< 0.001**
	Mild	366 (92.7%)	63 (65.6%)	
	Moderate	26 (6.6%)	30 (31.2%)	
	Severe	3 (0.8%)	3 (3.1%)	
Arteriolar smooth muscle vacuolar degeneration	None	113 (81.9%)	26 (70.3%)	**0.040**
	Mild	19 (13.8%)	11 (29.7%)	
	Moderate	6 (4.3%)	0 (0.0%)	
	Severe	0 (0.0%)	0 (0.0%)	

### Predictor selection

**Fig. 1:**
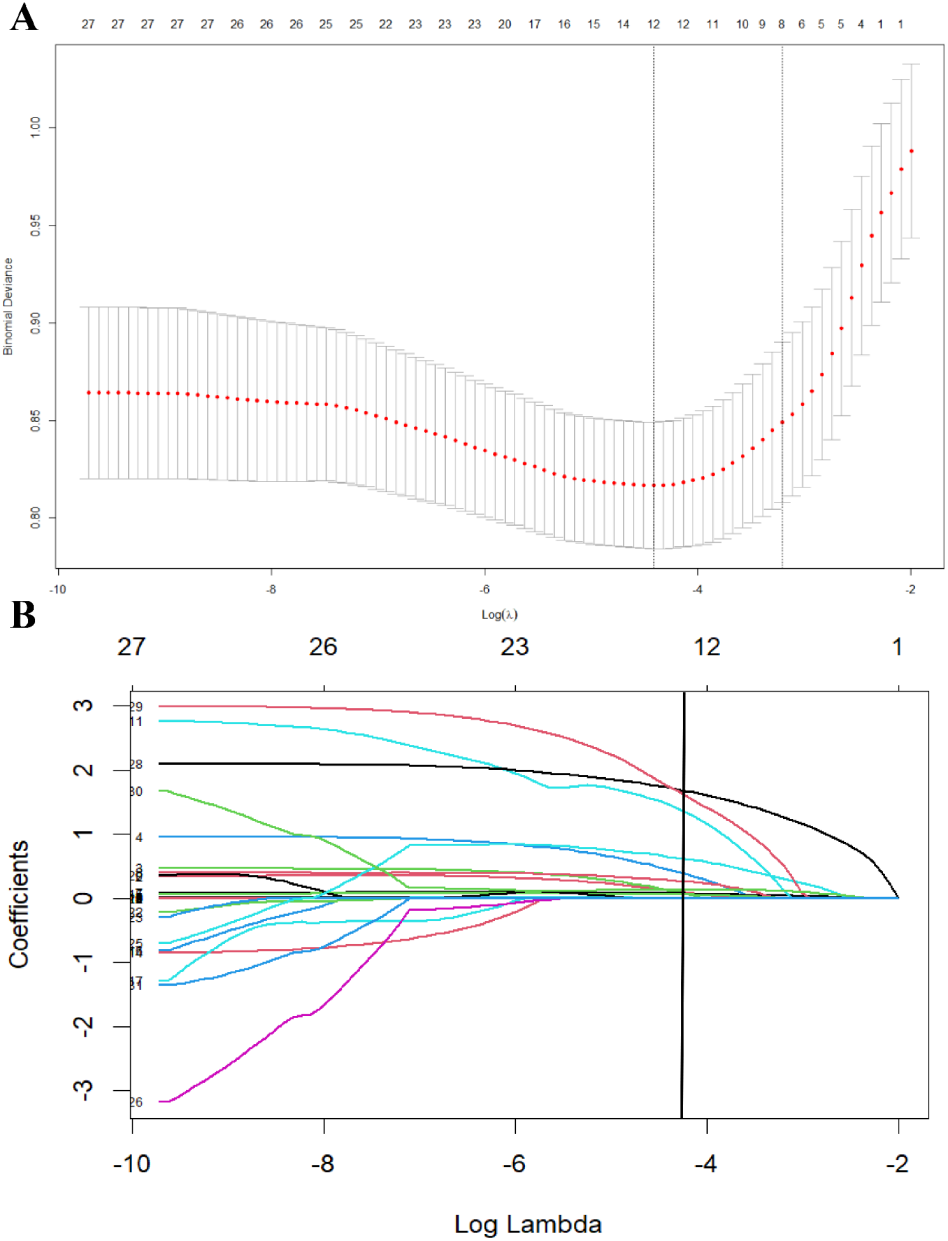
Variable selection using the least absolute shrinkage and selection operator (LASSO) binary logistic regression model. (**A**) Tuning parameter (λ) selection in the LASSO model used 10-fold cross-validation with the minimum criteria. The binomial deviance was plotted versus log(λ). Dotted vertical lines were drawn at the optimal values by using the minimum criteria and the 1-standard error of the minimum criteria (the 1-SE criteria). A λ value of 0.012, with log (λ), -4.423 was chosen (1-SE criteria) according to 10-fold cross-validation. (**B**) LASSO coefficient profiles of the 28 variables. A coefficient profile plot was produced against the log(λ) sequence. A vertical line was drawn at the value selected using 10-fold cross-validation, where optimal λ resulted in 12 nonzero coefficients (except for the intercept)

Twenty-eight variables measured before transplantation surgery were included in the LASSO regression model. After LASSO regression selection, 12 variables remained as potential predictors (Fig. 1A and B) and were features with nonzero coefficients in the LASSO logistic regression model. These 12 predictors included six donor characteristics (death reason, hypotensive procedure, CPR, BUN, SCr, and hypertension history), two organ preservation variables (initial perfusion resistance and WIT), and four pre-transplant biopsy features (mesangial matrix hyperplasia, moderate and severe ATI, and Banff score). Because of the high relevance of the relationship between terminal BUN and SCr levels, SCr was eliminated from the list of predictors to avoid multicollinearity. Severe ATI was integrated with moderate ATI because of its small sample size. Therefore, 12 variables with nonzero coefficients in the LASSO logistic regression model were reduced to 10 variables.

Table 5 shows the variables and their regression coefficients, LASSO-derived multivariate ORs, and the intercept of the model. The model identified CPR, terminal BUN level, and ATI as independent predictors.

**Table 5:** Variables selected by LASSO regression for predicting DGF

Variables	β	P value	OR (95%CI)	VIF
**Donor variables**				
Died of CVA or HIE	0.242	0.556	1.27 (0.57–2.87)	1.777
Hypotensive procedure (SBP < 100 mmHg)	0.447	0.163	1.56 (0.83–2.94)	1.185
CPR procedure	0.906	**0.041**	2.47 (1.01–5.84)	1.051
Terminal BUN (mmol/L)	0.112	**< 0.001**	1.12 (1.06–1.18)	1.849
Hypertension history	0.425	0.283	1.53 (0.71–3.38)	1.136
**HMP parameters**				
Initial perfusion resistance (mmHg·min·mL^− 1^)	2.019	0.087	7.54 (0.72–77.11)	1.068
**WIT (min)** **CIT (h)**	0.078 0.369	0.217 0.07	1.08 (0.95–1.23) 1.05 (0.97–1.11)	1.051 1.124
**Pre-transplant biopsies**				
Mesangial matrix hyperplasia	0.706	0.052	2.03 (0.99–4.15)	1.586
Moderate or severe ATI	2.131	**< 0.001**	8.42 (4.22–17.22)	1.059
Banff score	0.122	0.136	1.13 (0.96–1.33)	1.962

β is the regression coefficient. Terminal BUN, initial perfusion resistance, WIT, and Banff score were entered into the logistic model as continuous variables, and the other variables were entered as dichotomous variables. Abbreviations: CVA, cerebrovascular accident; HIE, hypoxic encephalopathy; SBP, systolic pressure; CPR, cardiopulmonary resuscitation; BUN, blood urea nitrogen; HMP, hypothermia machine perfusion; WIT, warm ischemia time; CIT, Cold ischemia time; ATI, acute tubular injury; VIF, variance inflation factor

### Construction of the preoperative DGF risk score

**Fig. 2:**
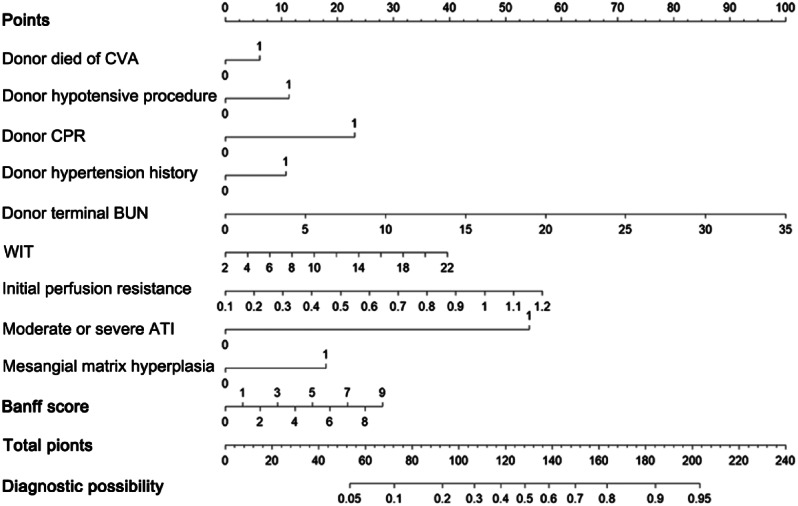
The developed preoperative DGF prediction nomogram. The nomogram was constructed using the data from the development cohort. Ten variables with nonzero coefficients selected by LASSO regression were presented. Terminal BUN level, initial perfusion resistance, WIT, and Banff score were entered into the logistic model as continuous variables, while the other variables were entered as dichotomous variables

The DGF risk score was constructed on the basis of the coefficients from the logistic model. We used the following formulas for the logistic model to calculate the probability and 95% CIs [18]: probability = exp(∑β × X)/[1 + exp(∑β × X)]; lower limit of 95% CI = exp[∑Xn × βn-∑z × SE(β)]/{1 + exp[∑Xn × βn-∑z × SE(β)]}; and upper limit of 95% CI = exp[∑Xn × βn + ∑z × SE(β)]/{1 + exp[∑Xn × βn + ∑z × SE(β)]}. The model that incorporated the above predictors was developed and presented as a nomogram (Fig. 2).

### Performance of the preoperative DGF risk score

**Fig. 3:**
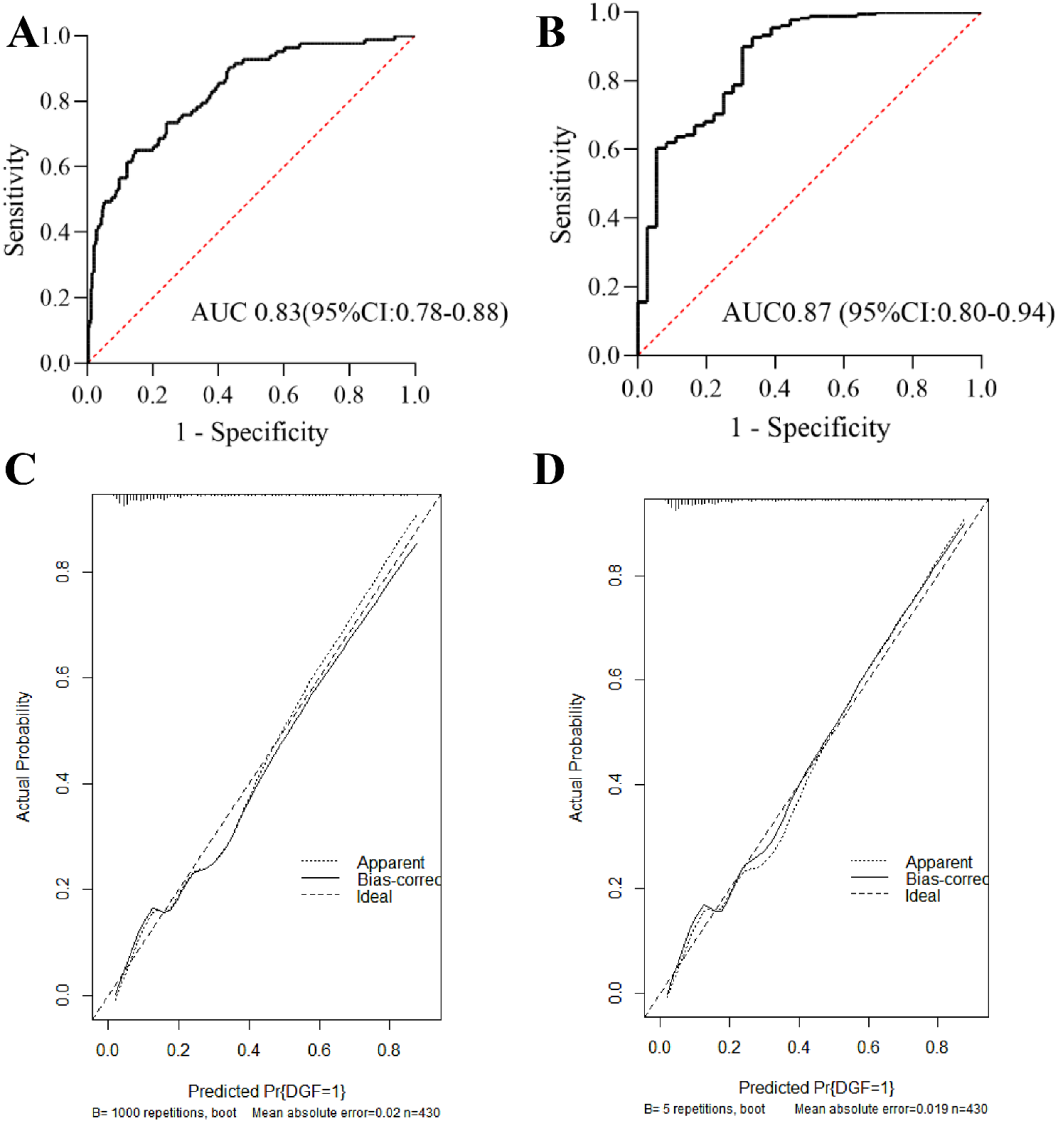
Predictive performance of the preoperative DGF prediction nomogram. (**A**) and (**B**) show the ROC curves of the nomogram in the development and validation cohorts, respectively. (**C**) and (**D**) present the calibration curves of the nomogram in the development and validation cohort, respectively. The calibration curve shows the calibration of the nomogram in terms of the agreement between the predicted risk of DGF and the observed risk of DGF. The 45° dotted line represents a perfect prediction, and the solid line represents the predictive performance of the nomogram. The solid line shows a closer fit to the dotted line, which indicates better predictive accuracy of the nomogram

The mean AUC values based on the data from the development and validation cohorts were 0.83 (95% CI, 0.78–0.88) and 0.87 (95% CI, 0.80–0.94), respectively (Fig. 3A and B). The calibration curve of the nomogram for DGF prediction demonstrated good agreement between the predicted and observed values in the development and validation cohorts (Fig. 3C and D). The H-L test yielded a nonsignificant statistical value (*P* = 0.712 and *P* = 0.509).

### Clinical application

**Fig. 4:**
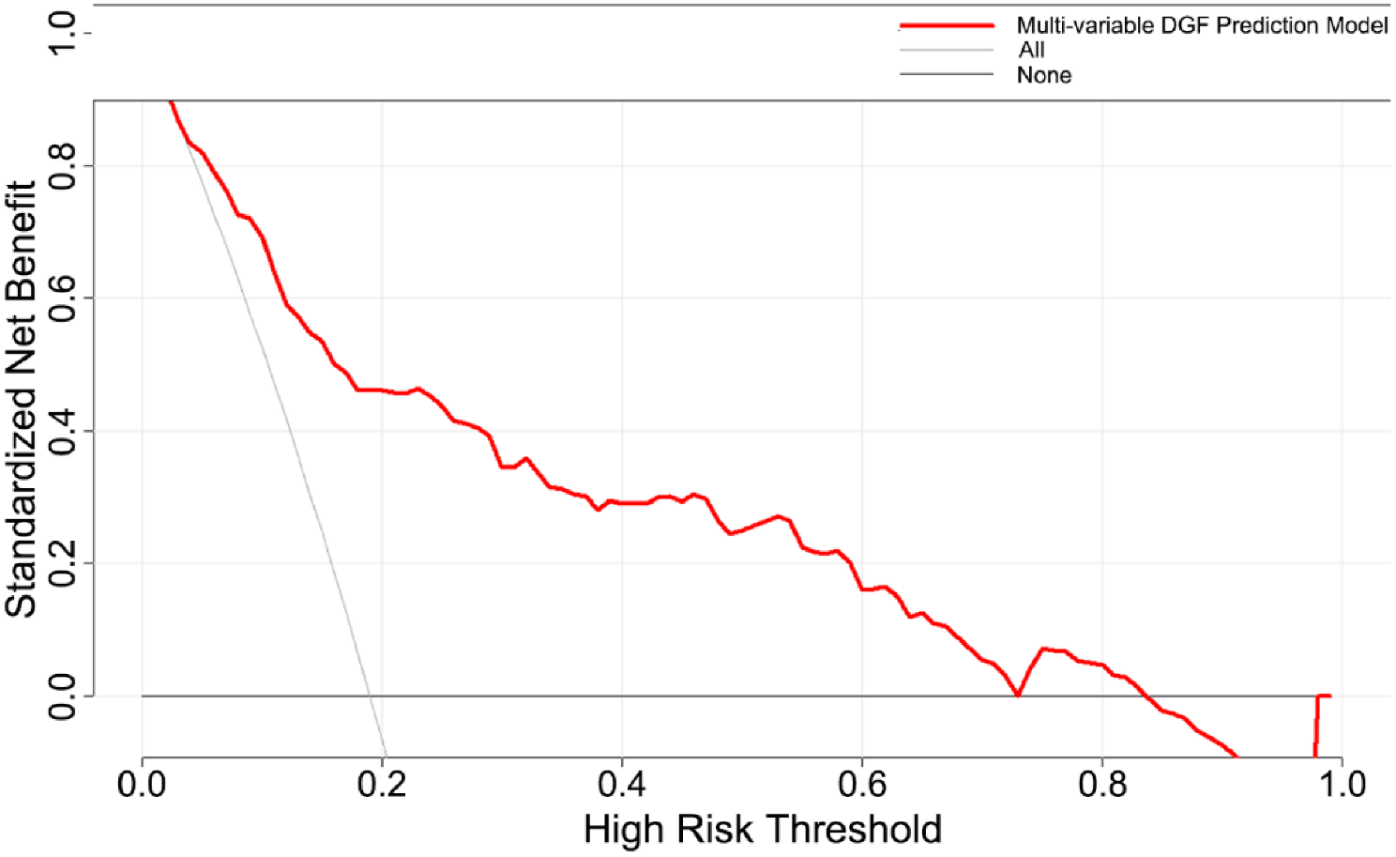
Decision curve analysis for the DGF prediction nomogram. The y-axis measures the net benefit. The red line represents the DGF prediction nomogram. The gray line represents the assumption that all recipients have DGF. The black line represents the assumption that no recipients have DGF. The net benefit was calculated by subtracting the proportion of all patients who are false positive from the proportion of those who are true positive, weighting by the relative harm of forgoing treatment when compared with negative consequences of an unnecessary treatment. The decision curve showed that if the threshold probability of a patient or doctor is > 5% and < 70%, the use of the nomogram in the current study to predict DGF adds more benefit than that achieved with the treat-all-patients scheme or the treat-none scheme

Figure 4 presents the DCA for the DGF prediction nomogram. The decision curve showed that if the threshold probability of a recipient is > 5% and < 70%, the use of the nomogram to predict DGF adds more benefit than that achieved with either the treat-all-patients scheme or the treat-none scheme.

## Discussion

Precise and early detection of recipients with a high risk of developing DGF before transplantation surgery is crucial as DGF is associated with adverse outcomes [19]. First, the occurrence of DGF increases the risk of acute rejection and long-term graft loss [20]. Second, DGF prolongs hospitalization, resulting in additional financial burden [20]. In a 5-year study of patients following kidney transplantation, early re-hospitalization after transplantation was a common event, occurring in 32% of the cohort, and only a few (9%) of these events had evidence of prevention. The leading causes of readmissions included surgical complications (15%), rejection (14%), volume metastasis (11%), and systemic and surgical wound infections (11% and 2.5%, respectively). Only 19 cases of rehospitalization (8%) met the criteria for prevention. The causes of early rehospitalization were varied, and the quality index after renal transplantation was also low [21].

Presently, the pathogenetic mechanisms of DGF remain unknown, and it is generally believed that both immune and nonimmune factors promote the occurrence and development of DGF [22]. These factors include donor-related factors (ischemia duration, age, kidney donor profile index [KDPI], end-stage sCr, history of hypertension, and CPR history), receptor-related factors (immune response, ischemia-reperfusion injury, and dialysis duration), and surgery-related factors [23]. The combined effect of these factors increases the allogenic response of organs, thus affecting their long-term survival. On the basis of etiology, predisposition, and underlying mechanisms, it is reasonable to infer that there are different subtypes of DGF, and consequently, the prognosis varies [24]. A recent study [25] reported commonly accepted risk factors for DGF, which included donation after cardiac death (DCD) donors, long CIT, long-distance transportation, pre-transplant dialysis recipients, past transplant recipients, diabetic recipients and higher BMI, longer waiting time, and donor-recipient mismatch. DGF is associated with an increased incidence of acute rejection and with poor long-term transplant outcomes. It is also associated with a lower quality of life. DGF leads to an increased financial burden; in a study based on the Premier Healthcare database, DGF was found to be associated with an average cost increase of approximately $18,000, an additional 6-day stay in the hospital, and an additional 2-day stay in the intensive care unit. Finally, together with the financial burden on the healthcare system, patients with DGF are also socially and psychologically affected, as they are often overwhelmed by the frequency of outpatient visits and their absence from home.

Currently, the accurate prediction of DGF is difficult to achieve; however, it is possible to identify high-risk DGF recipients at an early stage on the basis of a range of clinical and laboratory indicators. Our study included pathological and clinical data of 492 deceased donor (DD) kidney transplantation cases. The variables were selected by LASSO regression, and the multivariate logistic regression model with DGF as the endpoint was finally established. The nomogram of the model was drawn, and the practicability of the prediction model for evaluating donor kidney quality was assessed and verified.

Donor and recipient characteristics have significant implications for DGF prediction. In the last decade, Irish 2010 [9], Nyberg 2001 [26], Jeldres 2009 [19], and Chapal 2014 [27] conducted studies on the DGF predictive model. The variables included in each scoring system were different. The main DGF risk factors reported in these studies included donor age, BMI, terminal SCr level, cause of death, hypertension history, diabetes history, WIT, CIT, recipient BMI, HLA mismatch, PRA level, dialysis method and duration, and induction therapy method. In the present study, we extracted these risk factors and analyzed their correlation with DGF through the univariate analysis.

In our present study, CIT, recipient BMI, HLA mismatch, and dialysis method and duration were not correlated with DGF occurrence, mainly because of the following reasons. The recipients in China had a shorter waiting time before transplantation surgery. According to the 2015 Annual Chronic Kidney Disease Report in China [28], the median waiting time of domestic patients with uremia was 17.53 months, and approximately 40% of the recipients received kidney transplant surgery within 1 year after entering the waiting list. However, the median waiting time in USA for kidney transplant was 49.2 months [29]. Relatively, patients with uremia in China have shorter waiting time for kidney transplant and shorter dialysis time before transplantation. Uremia is a wasting disease. Longer waiting time and dialysis duration will deteriorate patients’ condition and decrease their tolerance to surgery, thereby resulting in a high risk of postoperative complications such as DGF and PNF. The variations in national and demographic characteristics can explain the differential risk factors of DGF to some extent.

When constructing the predictive model, it is worth noting that the effect of CIT on DGF is not significant, contrary to some previous studies [30]. In recent years, with the advancement of medicine and the deepening of DGF research, some researchers have found that the influence of CIT on DGF is gradually diminishing. For example, the survival rates of animal models of allografts treated with hydrogen sulfide in cold storage were significantly improved compared to controls (*P* < 0.01) [31]. In a large clinical database, also found that recipients using immunosuppressants after KT could also disregard the effects of CIT [27]. In analyzing data on 90,810 recipients of DD in the United States from 2010 to September 2018, reported that the risk of CIT for DGF was not significant [27]. Advances in the preservation of donor kidneys during transport, such as LifePort cryogenic machine perfusion, have significantly reduced the impact of CIT on renal ischemia-reperfusion injury [32]. Therefore, the series of trials and clinical cohort analyses described above suggest that central CIT has little significance for predicting DGF effects. In addition, using the original database, we found that the gender ratios of donors and recipients were highly imbalanced. This is related to the insufficient coverage of social ideology, family income and hospital propaganda. This imbalance will lead to significant errors in the model.

Previous studies have shown that HMP is beneficial and leads to significantly lower risk of DGF [33]. However, in the present study, no significant difference in DGF risk was observed between HMP-preserved donor kidneys and SCS-preserved donor kidneys; this might be due to the small number of SCS-preserved donor kidneys. The present study analyzed the correlation between HMP parameters and DGF. The results showed that the initial and terminal perfusion pressure, flow rate, and resistance parameters were significantly correlated with DGF; however, the difference in various parameters before and after perfusion was not significantly correlated with DGF. This study included a larger number of patients than a previous study and added the influence of Banff score and HMP on results. This finding was consistent with the results of previous research [8, 34–36].

Most chronic and acute histologic lesions in pre-transplant biopsy are independent risk factors of DGF; however, DGF predictive models have rarely included these histologic factors. The most important and final argument for using the nomogram is based on the combination of pre-transplant biopsies with donor clinical characteristics and HMP parameters. By performing variable selection through LASSO regression, this nomogram was significantly better than previous models reported in the above-mentioned literature [8, 34, 35]. The AUC values of this nomogram were 0.83 and 0.87 in the development and validation cohorts, respectively. These results demonstrate the potential utility of this model to predict patients at risk of developing DGF. The old model was compared with the current nomogram model to reflect the current era of kidney transplantation. The study population was refined to improve the prediction accuracy of the model, which can also be used to predict long-term graft survival before transplantation.

The established DGF risk prediction model derived and validated a potential clinical prediction tool rather than a decision rule. In this study, chronic and acute lesions in pretransplant biopsies were assessed by semi-quantitative methods. There were significant differences in the distribution of chronic and acute lesions between the DGF and non-DGF groups, except for arterial hyaline degeneration. The pre-transplantation biopsy score can be used to inform clinicians’ evidence-based decision making regarding the use of kidneys to guide the management of kidney transplantation. For example, based on the mildly impaired and moderately injured, we recommend that the DCD kidney can be used with minimal risk of DGF. However, at severe damage, we recommend being cautious in the application of the DCD kidney and should be used in specific clinical situations. The DGF risk prediction model established in this study has reference value for the selection of kidney transplant donors and can be used to predict DGF before organ donation acquisition.

The limitations of the present study include small sample size and single center study. For better disease prediction, the sample size should be increased to increase the accuracy of prediction. Additionally, future studies should include multiple centers to further validate the clinical application of this nomogram.

## Conclusion

In conclusion, this study presents a DGF prediction nomogram that incorporates donor clinical characteristics, HMP parameters, and pre-transplant biopsy features and can be conveniently used for preoperative individualized prediction of DGF in recipients before kidney transplantation surgery.

### Electronic supplementary material

Below is the link to the electronic supplementary material.

Supplementary Material 1

## Data Availability

The datasets generated and analysed during the current study are not publicly available due the datasets are private to the patients and the institution but are available from the corresponding author on reasonable request.

## Code Availability

R code can be available by contacting authors.
