# Correction: Development and validation of a novel nomogram model for predicting delayed graft function in deceased donor kidney transplantation based on pre-transplant biopsies

**DOI:** 10.1186/s12882-024-03641-8

**Published:** 2024-06-18

**Authors:** Meihe Li, Xiaojun Hu, Yang Li, Guozhen Chen, Chen-guang Ding, Xiaohui Tian, Puxun Tian, Heli Xiang, Xiaoming Pan, Xiaoming Ding, Wujun Xue, Jin Zheng

**Affiliations:** Department of Renal Transplantation, Nephropathy Hospital, The First Affiliated Hospital of Xi’an Jiaotong University, Xi’an, Shaanxi 710061 China

**Correction: BMC Nephrology (2024) 25:138** 10.1186/s12882-024-03557-3

Following publication of the original article [1], the authors identified that there are two duplicate names in the author’s information, Chen-guang Ding and Chenguang Ding are the same person. We have deleted Chenguang Ding.

The original article has been corrected.
