# Supplementary material for: Development and validation of a novel nomogram model for predicting delayed graft function in deceased donor kidney transplantation based on pre-transplant biopsies
Source: BMC Nephrol. 2024 Apr 19;25:138. doi: 10.1186/s12882-024-03557-3 (PMC11031976; doi:10.1186/s12882-024-03557-3)
Supplement: Supplementary file 1 — Supplementary Material 1 [file 12882_2024_3557_MOESM1_ESM.docx]

Table S1. Histopathological evaluation system of chronic lesions in pre-implantation biopsies

| Variables | Points | Contains |
| --- | --- | --- |
| Glomerular sclerosis | 0 | none globally sclerosed |
|  | 1 | <20% global glomerulosclerosis |
|  | 2 | 20 to 50% global glomerulosclerosis |
|  | 3 | >50% global glomerulosclerosis |
| Tubular atrophy | 0 | absent |
|  | 1 | <20% of tubule affected |
|  | 2 | 20 to 50% of tubule affected |
|  | 3 | >50% of tubule affected |
| Interstitial fibrosis | 0 | absent |
|  | 1 | <20% of renal tissue replaced by fibrous connective tissue |
|  | 2 | 20 to 50% of renal tissue |
|  | 3 | >50% of renal tissue replaced by fibrous connective tissue |
| Arterial and arteriolar narrowing | 0 | absent |
|  | 1 | increased wall thickness but to a degree that is less than the lumen |
|  | 2 | wall thickness that is equal or slightly greater to the diameter |
|  | 3 | wall thickness that far exceeds the diameter of the lumen with narrowing or occlusion |
| Glomerular mesangial matrix hyperplasia | 0 | absent |
|  | 1 | mesangial matrix hyperplasia occurs in <25% of the nonsclerosing glomeruli |
|  | 2 | mesangial matrix hyperplasia occurs in 25-50% of the nonsclerosing glomeruli |
|  | 3 | mesangial matrix hyperplasia occurs in >50% of the nonsclerosing glomeruli |
| Arterial hyaline degeneration | 0 | absent |
|  | 1 | arterial hyaline degeneration occurs in 1 arteriole branch |
|  | 2 | arterial hyaline degeneration occurs in 2-3 arteriole branches |
|  | 3 | arterial hyaline degeneration occurs in >3 arteriole branches |
| Banff score=glomerular sclerosis+tubular atrophy+interstitial fibrosis+arterial and arteriolar narrowing+glomerular mesangial matrix hyperplasia | | |
| Remuzzi score=glomerular sclerosis+tubular atrophy+interstitial fibrosis+arterial and arteriolar narrowing | | |

Table S2 Recipient characteristics comparison between DGF and non DGF group in validation cohort

| Recipient Characteristics | Non-DGF Group | DGF Group | *P* value |
| --- | --- | --- | --- |
|  | n=84 | n=21 |  |
| Age (years) | 36.62±9.44 | 36.38±10.35 | 0.924 |
| Male, n (%) | 58 (69.0%) | 15 (71.4%) | 0.999 |
| BMI (kg/m^2^) | 20.77±3.26 | 20.76±3.16 | 0.990 |
| Primary disease, n (%) |  |  | 0.999 |
| Chronic glomerulonephritis | 60 (71.4%) | 16 (76.2%) |  |
| Others | 24 (28.6%) | 5 (23.8%) |  |
| Hemodialysis, n (%) | 72 (85.7%) | 18 (85.7%) | 0.999 |
| Dialysis duration (months) | 19.28±19.50 | 22.01±24.10 | 0.634 |
| HLA mismatches | 2.0 (1.0, 2.0) | 2.0 (1.0, 2.0) | 0.328 |
| PRA positive, n (%) | 12 (14.3%) | 1 (4.8%) | 0.457 |

Data were presented as mean±SD, n (%) or median (interquartile range). DGF, delayed graft function; BMI, body mass index; HLA, human leukocyte antigen; PRA, panel reactive antibody.
